# Gross ways to live long: Parasitic worms as an anti-inflammaging therapy?

**DOI:** 10.7554/eLife.65180

**Published:** 2021-02-02

**Authors:** Bruce Zhang, David Gems

**Affiliations:** Institute of Healthy Ageing, and Research Department of Genetics, Evolution and Environment, University College London London United Kingdom; University of Geneva Switzerland; University of Geneva Switzerland

**Keywords:** evolutionary medicine, aging, inflammaging, hygiene hypothesis, old friends, helminth therapy

## Abstract

Evolutionary medicine argues that disease can arise because modern conditions do not match those in which we evolved. For example, a decline in exposure to commensal microbes and gastrointestinal helminths in developed countries has been linked to increased prevalence of allergic and autoimmune inflammatory disorders (the hygiene hypothesis). Accordingly, probiotic therapies that restore ‘old friend’ microbes and helminths have been explored as Darwinian treatments for these disorders. A further possibility is that loss of old friend commensals also increases the sterile, aging-associated inflammation known as inflammaging, which contributes to a range of age-related diseases, including cardiovascular disease, dementia, and cancer. Interestingly, Crowe et al., 2020 recently reported that treatment with a secreted glycoprotein from a parasitic nematode can protect against murine aging by induction of anti-inflammatory mechanisms. Here, we explore the hypothesis that restorative helminth therapy would have anti-inflammaging effects. Could worm infections provide broad-spectrum protection against age-related disease?

## Introduction

The aging process is the main cause of senescent multimorbidity, the co-occurrence of multiple chronic pathologies that includes the major diseases of late life. The geroscience approach views preventative intervention in the aging process as a means to simultaneously pre-empt the development of multiple age-related diseases (Austad, 2016). How does aging cause senescent multimorbidity? While there are clearly multiple contributory factors, one determinant whose importance is becoming increasingly clear is inflammaging, the state of systemic, low-grade inflammation that increases with age, independent of attack by infectious pathogens (Franceschi et al., 2000). Such inflammation is a contributory factor in diverse age-related pathologies, including cardiovascular disease, dementia, cancer, chronic obstructive pulmonary disease (COPD), osteoporosis, age-related macular degeneration (Xia et al., 2016), and perhaps even symptom severity during SARS-CoV-2 (COVID-19) infections (Akbar and Gilroy, 2020).

One cause of inflammaging is gut dysbiosis, an imbalance in the composition of the intestinal microbiome. Such imbalance can manifest as loss of immunomodulatory microbial species and is exacerbated by pro-dysbiotic aspects of the modern lifestyle, including antibiotic usage and the so-called Western diet (Buford, 2017). One interpretation of the cause of such dysbiotic effects utilizes the ‘old friends’ hypothesis (derived from the hygiene hypothesis [Strachan, 1989]). This argues that the human immune system evolved optimal function in a dirtier world, including the presence of various microbes and helminth parasites, whose removal leads to pathogenic immunological hyperactivity (Rook et al., 2004). Gut dysbiosis certainly does contribute to allergic and autoimmune inflammation prior to aging (Kamada et al., 2013). One corollary of the old friends hypothesis is that restoration of old friend species should attenuate such inflammatory hyperfunction and reduce disease. Indeed, fecal microbiota transfer from healthy donors has been suggested as a potential anti-inflammaging therapy (Franceschi and Campisi, 2014). While the possible role of the microbiome in inflammaging has engaged the imagination of biogerontologists, little consideration has been given to a possible similar role in aging of the macrobiome – in particular, helminth parasites, which include flukes, tapeworms, and nematodes.

**Figure 1.**
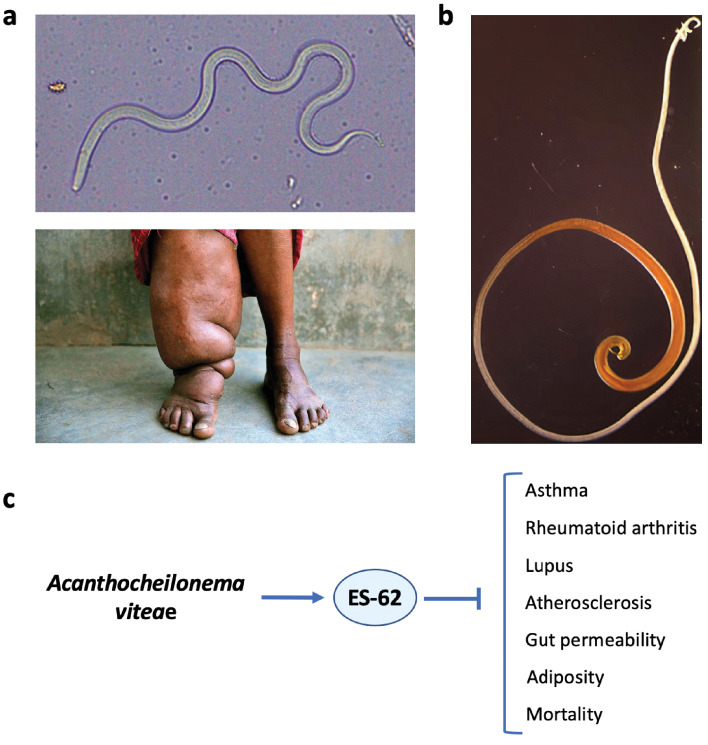
Helminth therapy to prevent Darwinian diseases of inflammatory hyperactivity. (**a**) Resistance is futile: the filarial nematode *Wuchereria bancrofti* (top) that causes elephantiasis in individuals with a hyperactive response to the parasite (bottom). (**b**) The whipworm *Trichuris suis*, a candidate for use in helminth therapy. (**c**) The rodent filarial worm *Acanthocheilonema viteae* secretes a glycoprotein, ES-62, which is protective in murine models of asthma, rheumatoid arthritis, lupus, and atherosclerosis. In addition, in a mouse model of high-calorie diet-accelerated aging, ES-62 administration prevented age-associated increases in gut permeability and adiposity, and increased longevity (Crowe et al., 2020).

**Figure 2.**
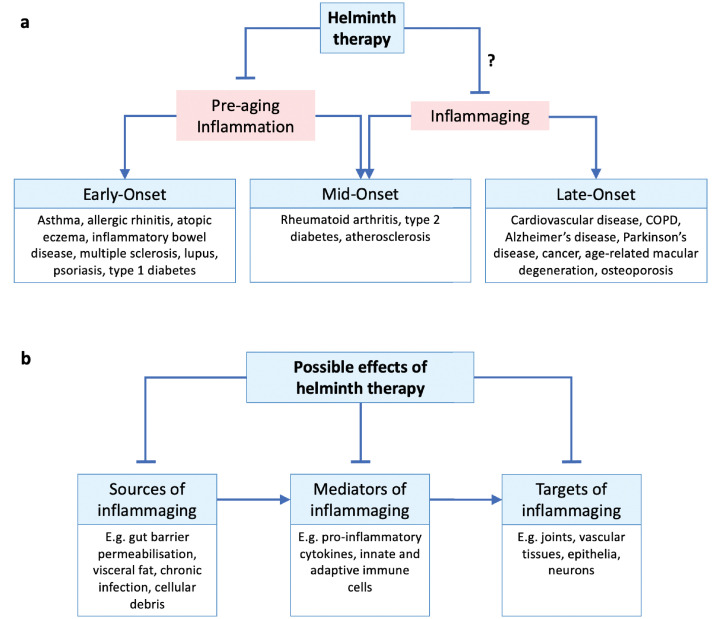
Helminth therapy and inflammaging. (**a**) Inflammatory disorders throughout the life course may be classed as early-, mid-, and late-onset disorders. Pre-aging inflammation (e.g. allergic and autoimmune inflammation) contributes to early- and some mid-onset disorders (all of which may persist into later life), while inflammaging appears later in life and contributes to late- and some mid-onset disorders. Helminth therapy has been demonstrated to protect against early- and mid-onset disorders by controlling pre-aging inflammation, but whether it can also protect against mid- and late-onset disorders through anti-inflammaging mechanisms remains uncertain. We note that MS (age of onset 20–50 years) and lupus (15–45 years) are sometimes described as inflammaging related (Sanai et al., 2016; Musella et al., 2018; Tsai et al., 2019). (**b**) Hypothetical scheme of helminth-mediated attenuation of inflammaging acting at different pathophysiological stages. ‘Sources of inflammaging’ are the mechanisms by which new inflammation is introduced; ‘mediators of inflammaging’ are the components of existing inflammation itself; ‘targets of inflammaging’ are the tissues damaged by the inflammation. Helminth therapy could in principle target the inflammatory source and/or mediator and/or the damaged tissues.

Parasitic helminths have infected humans throughout their evolutionary history. As a consequence, helminths have become master manipulators of our immune system in order to dodge host attack. Meanwhile, we have evolved some levels of tolerance of their presence. The wisdom of sometimes putting up with helminths is illustrated by the pathologies that can result from overly aggressive anti-helminth responses, as in elephantiasis that can result from infection with the filarial nematode *Wuchereria bancrofti* (Figure 1a). Through such coevolution, normal human immune development and function is likely to have become dependent upon the presence of immunomodulatory helminths, as well as that of their microbial counterparts (Velasquez-Manoff, 2012). Indeed, like old friend microbes, reduced helminth infection in ultra-clean modern societies has been linked to increased rates of allergic and autoimmune inflammatory disease, while restorative helminth therapies can protect against these conditions (see next section). But does reduced helminth infection, like gut dysbiosis, also promote inflammaging in later life? And, consequently, could restorative therapy with helminths, given at low doses that do not cause illness, reduce inflammaging, and ameliorate the pathologies that it promotes (Figure 2a)?

Using a mouse model, Crowe et al., 2020 recently provided the first tantalizing evidence that helminth therapy can protect against aging through anti-inflammatory mechanisms. In the light of their study, we have taken a conceptual research approach (surveying and repurposing published findings) (Blagosklonny and Pardee, 2002) to investigate the plausibility of anti-inflammaging helminth therapy. We will begin by describing the idea of helminth therapy in more detail.

### Helminth therapy as a treatment for putative old friend loss disorders

A variety of inflammatory disorders that afflict people prior to aging have been linked to a loss of old friend helminths. These include asthma, atopic eczema, inflammatory bowel disease, multiple sclerosis (MS), rheumatoid arthritis, and type 1 diabetes (Maizels, 2020). For example, a study from Argentina found that accidental acquisition of helminth infections not only caused a reduction in inflammatory cytokines, but also alleviated disease symptoms in MS patients, and that clearance of parasites reversed these effects (Correale and Farez, 2007; Correale and Farez, 2011). In a similar vein, studies from Uganda found that hookworm infections in pregnant women confer protection against infantile atopic eczema, protection that is abrogated by anthelmintic treatment during pregnancy (Mpairwe et al., 2011; Mpairwe et al., 2014).

More directly, a number of studies have documented beneficial effects of deliberate helminth infections, beginning with J.E. Turton's 1976 report in *The Lancet*, which describes how maintaining an infection with the intestinal hookworm *Necator americanus* alleviated his allergies (Turton, 1976). Helminth therapy (including treatment with individual parasite antigens) has also been explored extensively in rodents. For example, to date, 20 studies of the autoimmune encephalomyelitis mouse model of MS have reported helminth-induced reductions in disease severity (Charabati et al., 2020).

Unsurprisingly, the use of live helminths to treat old friend disorders remains controversial, and giving oneself worms seems unwholesome, to say the least. More seriously, there are obvious safety worries: live helminth therapy might cause a resurgence of harmful infections in de-wormed countries or induce harmful side effects. However, these concerns may be assuaged by controlling dose size and using helminths with different definitive hosts, such as the pig whipworm *Trichuris suis* which does not reproduce in humans (Figure 1b). However, although some initial clinical trials of *T. suis* ova therapy to treat patients with inflammatory bowel disease yielded promising results (Elliott and Weinstock, 2017), follow-up trials have not thus far (Huang et al., 2018). A possibility here is that only true old friends (i.e. anthropophilic helminths) will provide therapeutic benefits; notably, there is evidence that treatment with *N. americanus* hookworms is beneficial against celiac disease, an autoimmune disease affecting the bowel (Croese et al., 2015).

Another strategy is to identify the mechanisms by which helminths manipulate host immunity and then apply those therapeutically. For example, administration of the glycoprotein ES-62 secreted by the rodent filarial nematode *Acanthocheilonema viteae* can recapitulate many of the immunomodulatory changes resulting from filarial infections and is protective in several pathological contexts (Figure 1c). Similarly, a variety of helminth-derived proteins are protective in rodent models of MS (Dixit et al., 2017). Such approaches seem likely to yield safer and more precisely targeted therapeutics and are certainly more palatable. However, long-lasting, low-level helminth infections might still represent cheaper and more efficient forms of therapy, providing the full panoply of immunomodulatory agents and in a form that is easy to administer; c.f. ES-62 that was given in weekly injections (Crowe et al., 2020).

Taken together, helminth therapy currently shows promise as a means to treat inflammatory conditions such as allergy and autoimmunity (Figure 2a). But could it also protect against inflammaging? Could maintaining half a dozen hookworms in your bowel help slow aging?

### Helminth therapy to counter inflammaging?

Inflammaging can be detected as a sterile, persistent elevation of pro-inflammatory molecules in the blood, including cytokines such as IL-6 and TNF-α and acute phase proteins such as C-reactive protein (CRP). Interestingly, reduced levels of circulating pro-inflammatory cytokines and CRP have been seen in people infected by strongylid and filarial helminths in a number of studies (Aravindhan et al., 2010; George et al., 2014; Rajamanickam et al., 2019; Rajamanickam et al., 2020) (though not all [Aravindhan et al., 2012]), and experimental helminth infection similarly reduces pro-inflammatory cytokines in human serum (Gaze et al., 2012). Furthermore, expulsion of helminths by anthelmintic medication induces elevated pro-inflammatory cytokine responsiveness in human blood (Bourke et al., 2013; Wammes et al., 2016). Thus, it is possible that helminth infections could attenuate the systemic inflammation that is inflammaging. But what about actual benefits in terms of improved health outcomes, such as reduced pathology and increased lifespan?

ES-62 is a 62 kDa glycoprotein found in the excretory secretory products of the filarial nematode parasite *A. viteae* with anti-inflammatory properties. In the first study of its kind, Crowe et al., 2020 reported that weekly administration of ES-62 improved late-life health and increased lifespan (+12%, median lifespan) in a C57BL/6J mouse model of high-calorie diet-accelerated aging. This suggests that the anti-inflammatory capacities of helminth therapy can exert protective effects even in later life.

Administration of ES-62 prevented the age-related decline in gut barrier integrity seen in high-calorie diet-accelerated aging (Crowe et al., 2020). Of interest in the present context is that permeabilization of the gut lining can contribute to inflammaging, by allowing foreign antigens to leak into the blood and induce systemic immune activation (Thevaranjan et al., 2017; Ferrucci and Fabbri, 2018). But how could helminth therapy maintain gut barrier integrity? One way may be through the prevention of gut dysbiosis, a known contributor to inflammaging. Indeed, ES-62 prevented and, to some extent, even reversed age-related decreases in *Bacteroidetes*:*Firmicutes* ratio and increases in *Proteobacteria* in the gut (Crowe et al., 2020), both of which are associated with inflammaging (Fransen et al., 2017). Another possible mode of action is helminth-induced production of IL-22, an essential cytokine for maintaining gut barrier integrity (Sonnenberg et al., 2011). Interestingly, the intestinal murine roundworm *Heligmosomoides polygyrus* can even induce fetal-like reversion in intestinal cells to effect tissue repair (Nusse et al., 2018). Thus, it is plausible that the extended lifespan was due to suppression of intestinal changes that contribute to inflammaging.

Administration of ES-62 also prevented adipocyte hypertrophy in gonadal visceral fat (Crowe et al., 2020). This, again, is notable given that adiposity is a major source of inflammaging. Adipose tissue, particularly visceral fat, is highly infiltrated by immune cells, such as monocytes, B and T lymphocytes, mast cells, and neutrophils, and is also a major site of senescent cell accumulation (Tchkonia et al., 2010; Mraz and Haluzik, 2014). Together, these adipocytes, immune cells, and senescent cells secrete a potent and destructive cocktail of pro-inflammatory molecules, including TNF-α, IL-6, IFN-γ, and leptin, which enter systemic circulation and contribute to the inflammaging phenotype. At the same time, anti-inflammatory mechanisms are reduced in adipose tissues, as shown by reduced numbers of eosinophils and of adiponectin secretion. In line with this, ES-62 administration reduced the decline in eosinophil number in visceral fat (Crowe et al., 2020).

Thus, one potential mode of action of helminth therapy is suppression of adiposity. How could it achieve this? *Nippostrongylus brasiliensis*, a gastrointestinal roundworm of rodents, reduces adiposity apparently by reducing intestinal glucose absorption through altered STAT6 signaling (Moyat et al., 2019). Whether humans show similar responses is unclear. One study of humans reported an inverse correlation between previous schistosome infection and central obesity, though adjustment for socio-economic status was not performed (Shen et al., 2015). More generally, given the relationship between adiposity and inflammaging, helminth therapy might be of particular benefit to people who are overweight or obese.

The findings by Crowe et al., 2020 provide new support for the possibility that helminth therapy could protect against inflammaging. It would be interesting to know how ES-62 affects systemic markers of inflammation during inflammaging, which were not measured in the study, and also to examine effects on specific diseases linked to inflammaging. A number of earlier studies suggest that helminths can protect against such diseases, including rheumatoid arthritis (Ferrucci and Fabbri, 2018; Rea et al., 2018), type 2 diabetes (de Candia et al., 2019; Zuo et al., 2019), atherosclerosis (Zhuang and Lyga, 2014), and cancer (Leonardi et al., 2018; Zuo et al., 2019; Figure 2a). We will consider the evidence for each of these in turn.

A prediction of the ‘anti-inflammaging helminth hypothesis’ is that in areas endemic for helminth infection, there should be lower rates of inflammaging-related disease, and there is some evidence for this. For example, in a region of Eastern India endemic for lymphatic filariasis infection, it was found that not one of 207 rheumatoid arthritis patients tested positive for circulating filarial nematode antigens; by contrast, 40% of 222 healthy controls were antigen positive (Panda et al., 2013). This is consistent with a protective role of helminth infection against rheumatoid arthritis. Indeed, experimental helminth infection or treatment with helminth antigens can protect against pathology in the collagen-induced arthritis (CIA) and MRL/*lpr* mouse models of rheumatoid arthritis (Smallwood et al., 2017; Langdon et al., 2019). Notably, ES-62-mediated protection against CIA was accompanied by reduced serum levels of the pro-inflammatory cytokine IL-17 (Pineda et al., 2012), consistent with protection against systemic inflammation.

Broadly similar findings have been reported for type 2 diabetes and atherosclerosis. Several (but not all [Mendonça et al., 2006]) epidemiological studies have reported an inverse relationship between the incidence of helminth infection and type 2 diabetes (Aravindhan et al., 2010; Chen et al., 2013; Hays et al., 2015), and also serum levels of the pro-inflammatory cytokines IL-6 and GM-CSF (Aravindhan et al., 2010); these studies controlled for age and body mass index (and/or income). And again, experimental helminth infections protected mice from type 2 diabetes-associated states such as hyperglycemia and insulin resistance (Wu et al., 2011; Morimoto et al., 2016; Pace et al., 2018), while anthelmintic treatment elevated blood glucagon and insulin resistance, and several circulating pro-inflammatory cytokines (Tahapary et al., 2017; Rajamanickam et al., 2019; Rajamanickam et al., 2020). Regarding atherosclerosis, the burden of *Opisthorchis felineus* (cat liver fluke) in cadavers was found to be negatively correlated with the severity of aortic atherosclerosis (Magen et al., 2013). In atherosclerotic mouse models, infection with the blood fluke *Schistosoma mansoni* reduced atherosclerotic lesions by 50% (Doenhoff et al., 2002); similarly, administration of *Schistosoma* egg antigens reduced atherosclerotic plaque size (Zhang et al., 2012; Wolfs et al., 2014), and ES-62 reduced atherosclerotic lesion area by 60% in the *gld*.apoE^−/−^ mouse model of lupus-accelerated atherosclerosis (Aprahamian et al., 2015).

There is also some very limited evidence that helminth therapy might promote resistance to some forms of cancer. The tapeworm *Taenia crassiceps* and its antigens have been found to attenuate and prevent colon tumorigenesis in mice through anti-inflammatory mechanisms (León-Cabrera et al., 2014; Callejas et al., 2019). However, in the same mouse model, the nematode *H. polygyrus* was found to promote tumorigenesis (Pastille et al., 2017). Moreover, certain parasitic helminths are well known to increase cancer rates, for example the trematode parasite *Schistosoma haematobium* causes bladder cancer (Vennervald and Polman, 2009).

There is also some evidence linking helminth loss to other inflammaging-linked conditions. This includes COPD, given that some helminths are known to suppress lung inflammation and to reduce levels of the pro-inflammatory cytokine IL-33, a driver of COPD pathology (Osbourn et al., 2017). It should be noted however that other helminths such as *N. brasiliensis* can promote COPD pathology (Marsland et al., 2008). Meanwhile, IL-33 also promotes age-related macular degeneration (Xi et al., 2016), another late-life inflammaging-linked disease. Anecdotal evidence from home-users of live helminth therapy suggests that old friend helminths may also provide protection against Parkinson’s disease and depression, although whether the latter includes geriatric depression is not known (Cheng et al., 2015). Both Parkinson’s disease and geriatric depression are considered to be inflammaging-linked conditions (Teixeira et al., 2012; Calabrese et al., 2018).

### Perspectives

There is clear evidence that an absence of helminth infection leads to increased incidence of inflammatory disorders, including some inflammaging-linked conditions, such as rheumatoid arthritis. Furthermore, experimental and clinical work has demonstrated the protective benefits of restorative helminth therapy against some inflammaging-linked diseases. Whether this reflects the ability of helminth therapy to reduce inflammaging remains uncertain, but this possibility clearly warrants further investigation.

Given the role of inflammaging in numerous diseases of aging, further studies examining the effect of helminth therapy on multimorbidity would be especially useful. For example, it would be helpful to extend ongoing helminth therapy trials aimed at individual diseases to look at a broader range of conditions. The anti-inflammaging potential of helminth therapy could also be assessed by monitoring pro-inflammatory cytokine levels following administration of helminth therapy in patients with detectable inflammaging. Where live helminth therapy is used, infections can be easily terminated by anthelmintics should the need arise; conveniently, this would also provide additional data on the therapy’s efficacy (i.e. does inflammaging then worsen?). Regarding ES-62 in particular, it would be worthwhile to test its effects on aging in mice maintained on a normal diet rather than on a life-shortening high-calorie diet.

The possibility of anti-aging helminth therapy raises various questions. How do responses to such therapy change with age? Would higher or lower parasite (or antigen) levels be needed to take account of immunosenescence? To what extent do risks of helminth therapy increase in old age? What are optimal ages to apply such therapy to reduce inflammaging? Would helminth therapy act only in a preventative fashion (typical of anti-aging treatments), delaying the genesis or worsening of inflammaging-linked pathologies, or could it reverse existing disease symptoms? At least for murine rheumatoid arthritis, there is some evidence that helminth therapy could be both prophylactic and therapeutic (McInnes et al., 2003; Rzepecka et al., 2015). Thus, it is possible that helminth therapy could, to some extent, reverse symptoms of aging as well as decelerate them. Would responses to helminth therapy differ between the sexes? Here it is notable that ES-62 extended lifespan in male, but not female obese mice (Crowe et al., 2020). A further possibility is that helminths might inhibit tissue aging in their hosts in order to protect their local niche; notably, the pro-aging mTOR pathway was found to be inhibited in human dendritic cells by *Brugia malayi* microfilariae (Narasimhan et al., 2016).

Effective application of helminth therapy would also require a clear understanding of its specific anti-inflammaging mechanisms. Theoretically, helminths could counter inflammaging in several ways. For example, they could inhibit sources of inflammaging by preventing gut barrier permeabilization and obesity, neutralize existing inflammaging by increasing the proportion of anti-inflammatory to pro-inflammatory cytokines, or repair inflammaging-afflicted tissue damage, for example through promotion of IL-22 secretion (Figure 2b).

### Conclusions

It goes without saying that improvements in hygiene and elimination of helminth parasites have been of incalculable benefit to humanity. But a cost coupled with this benefit is abnormalities of immune function, specifically inflammatory hyperfunction. Available evidence suggests that restorative helminth therapies are effective against not only allergic and autoimmune inflammatory disorders, but also age-associated inflammation in later life, at least to some extent. Should this be confirmed, helminth therapy could provide protection against the wide spectrum of age-related diseases promoted by inflammaging. In the wake of successes during the last century in eliminating the evils of helminth infections, the time now seems propitious to explore further their possible benefits, particularly for our aging population – strange though this may sound.

## References

[bib1] Akbar AN, Gilroy DW (2020). ‘Aging immunity may exacerbate COVID-19’. Science.

[bib2] Aprahamian TR, Zhong X, Amir S, Binder CJ, Chiang LK, Al-Riyami L, Gharakhanian R, Harnett MM, Harnett W, Rifkin IR (2015). The immunomodulatory parasitic worm product ES-62 reduces lupus-associated accelerated atherosclerosis in a mouse model. International Journal for Parasitology.

[bib3] Aravindhan V, Mohan V, Surendar J, Muralidhara Rao M, Pavankumar N, Deepa M, Rajagopalan R, Kumaraswami V, Nutman TB, Babu S (2010). Decreased prevalence of lymphatic filariasis among diabetic subjects associated with a diminished pro-inflammatory cytokine response (CURES 83). PLOS Neglected Tropical Diseases.

[bib4] Aravindhan V, Surendar J, Mohan V, Rao MM, Deepa M, Anuradha R, Babu S (2012). Effect of filarial infection on serum inflammatory and atherogenic biomarkers in coronary artery disease (CURES-121). The American Journal of Tropical Medicine and Hygiene.

[bib5] Austad SN, Sierra F, Kohanski R (2016). Advances in Geroscience.

[bib6] Blagosklonny MV, Pardee AB (2002). Conceptual biology: unearthing the gems. Nature.

[bib7] Bourke CD, Nausch N, Rujeni N, Appleby LJ, Mitchell KM, Midzi N, Mduluza T, Mutapi F (2013). Integrated analysis of innate, Th1, Th2, Th17, and regulatory cytokines identifies changes in immune polarisation following treatment of human schistosomiasis. The Journal of Infectious Diseases.

[bib8] Buford TW (2017). (Dis)Trust your gut: the gut microbiome in age-related inflammation, health, and disease. Microbiome.

[bib9] Calabrese V, Santoro A, Monti D, Crupi R, Di Paola R, Latteri S, Cuzzocrea S, Zappia M, Giordano J, Calabrese EJ, Franceschi C (2018). Aging and Parkinson's Disease: Inflammaging, neuroinflammation and biological remodeling as key factors in pathogenesis. Free Radical Biology and Medicine.

[bib10] Callejas BE, Mendoza-Rodríguez MG, Villamar-Cruz O, Reyes-Martínez S, Sánchez-Barrera CA, Rodríguez-Sosa M, Delgado-Buenrostro NL, Martínez-Saucedo D, Chirino YI, León-Cabrera SA, Pérez-Plasencia C, Vaca-Paniagua F, Arias-Romero LE, Terrazas LI (2019). Helminth-derived molecules inhibit colitis-associated Colon cancer development through NF-κB and STAT3 regulation. International Journal of Cancer.

[bib11] Charabati M, Donkers SJ, Kirkland MC, Osborne LC (2020). A critical analysis of helminth immunotherapy in multiple sclerosis. Multiple Sclerosis Journal.

[bib12] Chen Y, Lu J, Huang Y, Wang T, Xu Y, Xu M, Li M, Wang W, Li D, Bi Y, Ning G (2013). Association of previous schistosome infection with diabetes and metabolic syndrome: a cross-sectional study in rural china. The Journal of Clinical Endocrinology & Metabolism.

[bib13] Cheng AM, Jaint D, Thomas S, Wilson JK, Parker W (2015). Overcoming evolutionary mismatch by Self-Treatment with helminths: current practices and experience. Journal of Evolutionary Medicine.

[bib14] Correale J, Farez M (2007). Association between parasite infection and immune responses in multiple sclerosis. Annals of Neurology.

[bib15] Correale J, Farez MF (2011). The impact of parasite infections on the course of multiple sclerosis. Journal of Neuroimmunology.

[bib16] Croese J, Giacomin P, Navarro S, Clouston A, McCann L, Dougall A, Ferreira I, Susianto A, O'Rourke P, Howlett M, McCarthy J, Engwerda C, Jones D, Loukas A (2015). Experimental hookworm infection and gluten microchallenge promote tolerance in celiac disease. Journal of Allergy and Clinical Immunology.

[bib17] Crowe J, Lumb FE, Doonan J, Broussard M, Tarafdar A, Pineda MA, Landabaso C, Mulvey L, Hoskisson PA, Babayan SA, Selman C, Harnett W, Harnett MM (2020). The parasitic worm product ES-62 promotes health- and life-span in a high calorie diet-accelerated mouse model of ageing. PLOS Pathogens.

[bib18] de Candia P, Prattichizzo F, Garavelli S, De Rosa V, Galgani M, Di Rella F, Spagnuolo MI, Colamatteo A, Fusco C, Micillo T, Bruzzaniti S, Ceriello A, Puca AA, Matarese G (2019). Type 2 diabetes: how much of an autoimmune disease?. Frontiers in Endocrinology.

[bib19] Dixit A, Tanaka A, Greer JM, Donnelly S (2017). Novel therapeutics for multiple sclerosis designed by parasitic worms. International Journal of Molecular Sciences.

[bib20] Doenhoff MJ, Stanley RG, Griffiths K, Jackson CL (2002). An anti-atherogenic effect of Schistosoma mansoni infections in mice associated with a parasite-induced lowering of blood total cholesterol. Parasitology.

[bib21] Elliott DE, Weinstock JV (2017). Nematodes and human therapeutic trials for inflammatory disease. Parasite Immunology.

[bib22] Ferrucci L, Fabbri E (2018). Inflammageing: chronic inflammation in ageing, cardiovascular disease, and frailty. Nature Reviews Cardiology.

[bib23] Franceschi C, Bonafè M, Valensin S, Olivieri F, De Luca M, Ottaviani E, De Benedictis G (2000). Inflamm-aging. an evolutionary perspective on immunosenescence. Annals of the New York Academy of Sciences.

[bib24] Franceschi C, Campisi J (2014). Chronic inflammation (Inflammaging) and its potential contribution to Age-Associated diseases. The Journals of Gerontology Series A: Biological Sciences and Medical Sciences.

[bib25] Fransen F, van Beek AA, Borghuis T, Aidy SE, Hugenholtz F, van der Gaast-de Jongh C, Savelkoul HFJ, De Jonge MI, Boekschoten MV, Smidt H, Faas MM, de Vos P (2017). Aged gut Microbiota contributes to systemical inflammaging after transfer to Germ-Free mice. Frontiers in Immunology.

[bib26] Gaze S, McSorley HJ, Daveson J, Jones D, Bethony JM, Oliveira LM, Speare R, McCarthy JS, Engwerda CR, Croese J, Loukas A (2012). Characterising the mucosal and systemic immune responses to experimental human hookworm infection. PLOS Pathogens.

[bib27] George PJ, Kumar NP, Sridhar R, Hanna LE, Nair D, Banurekha VV, Nutman TB, Babu S (2014). Coincident helminth infection modulates systemic inflammation and immune activation in active pulmonary tuberculosis. PLOS Neglected Tropical Diseases.

[bib28] Hays R, Esterman A, Giacomin P, Loukas A, McDermott R (2015). Does Strongyloides stercoralis infection protect against type 2 diabetes in humans? evidence from australian aboriginal adults. Diabetes Research and Clinical Practice.

[bib29] Huang X, Zeng LR, Chen FS, Zhu JP, Zhu MH (2018). Trichuris suis ova therapy in inflammatory bowel disease: a meta-analysis. Medicine.

[bib30] Kamada N, Seo SU, Chen GY, Núñez G (2013). Role of the gut Microbiota in immunity and inflammatory disease. Nature Reviews Immunology.

[bib31] Langdon K, Phie J, Thapa CB, Biros E, Loukas A, Haleagrahara N (2019). Helminth-based therapies for rheumatoid arthritis: a systematic review and meta-analysis. International Immunopharmacology.

[bib32] León-Cabrera S, Callejas BE, Ledesma-Soto Y, Coronel J, Pérez-Plasencia C, Gutiérrez-Cirlos EB, Ávila-Moreno F, Rodríguez-Sosa M, Hernández-Pando R, Marquina-Castillo B, Chirino YI, Terrazas LI (2014). Extraintestinal helminth infection reduces the development of colitis-associated tumorigenesis. International Journal of Biological Sciences.

[bib33] Leonardi GC, Accardi G, Monastero R, Nicoletti F, Libra M (2018). Ageing: from inflammation to Cancer. Immunity & Ageing.

[bib34] Magen E, Bychkov V, Ginovker A, Kashuba E (2013). Chronic Opisthorchis felineus infection attenuates atherosclerosis--an autopsy study. International Journal for Parasitology.

[bib35] Maizels RM (2020). Regulation of immunity and allergy by helminth parasites. Allergy.

[bib36] Marsland BJ, Kurrer M, Reissmann R, Harris NL, Kopf M (2008). Nippostrongylus brasiliensis infection leads to the development of emphysema associated with the induction of alternatively activated macrophages. European Journal of Immunology.

[bib37] McInnes IB, Leung BP, Harnett M, Gracie JA, Liew FY, Harnett W (2003). A novel therapeutic approach targeting articular inflammation using the filarial nematode-derived phosphorylcholine-containing glycoprotein ES-62. The Journal of Immunology.

[bib38] Mendonça SC, Gonçalves-Pires MR, Rodrigues RM, Ferreira A, Costa-Cruz JM (2006). Is there an association between positive Strongyloides stercoralis serology and diabetes mellitus?. Acta Tropica.

[bib39] Morimoto M, Azuma N, Kadowaki H, Abe T, Suto Y (2016). Regulation of type 2 diabetes by helminth-induced Th2 immune response. Journal of Veterinary Medical Science.

[bib40] Moyat M, Coakley G, Harris NL (2019). The interplay of type 2 immunity, helminth infection and the Microbiota in regulating metabolism. Clinical & Translational Immunology.

[bib41] Mpairwe H, Webb EL, Muhangi L, Ndibazza J, Akishule D, Nampijja M, Ngom-wegi S, Tumusime J, Jones FM, Fitzsimmons C, Dunne DW, Muwanga M, Rodrigues LC, Elliott AM (2011). Anthelminthic treatment during pregnancy is associated with increased risk of infantile eczema: randomised-controlled trial results. Pediatric Allergy and Immunology.

[bib42] Mpairwe H, Ndibazza J, Webb EL, Nampijja M, Muhangi L, Apule B, Lule S, Akurut H, Kizito D, Kakande M, Jones FM, Fitzsimmons CM, Muwanga M, Rodrigues LC, Dunne DW, Elliott AM (2014). Maternal hookworm modifies risk factors for childhood eczema: results from a birth cohort in Uganda. Pediatric Allergy and Immunology.

[bib43] Mraz M, Haluzik M (2014). The role of adipose tissue immune cells in obesity and low-grade inflammation. Journal of Endocrinology.

[bib44] Musella A, Gentile A, Rizzo FR, De Vito F, Fresegna D, Bullitta S, Vanni V, Guadalupi L, Stampanoni Bassi M, Buttari F, Centonze D, Mandolesi G (2018). Interplay between age and neuroinflammation in multiple sclerosis: effects on motor and cognitive functions. Frontiers in Aging Neuroscience.

[bib45] Narasimhan PB, Bennuru S, Meng Z, Cotton RN, Elliott KR, Ganesan S, McDonald-Fleming R, Veenstra TD, Nutman TB, Tolouei Semnani R (2016). Microfilariae of Brugia malayi inhibit the mTOR pathway and induce autophagy in human dendritic cells. Infection and Immunity.

[bib46] Nusse YM, Savage AK, Marangoni P, Rosendahl-Huber AKM, Landman TA, de Sauvage FJ, Locksley RM, Klein OD (2018). Parasitic helminths induce fetal-like reversion in the intestinal stem cell niche. Nature.

[bib47] Osbourn M, Soares DC, Vacca F, Cohen ES, Scott IC, Gregory WF, Smyth DJ, Toivakka M, Kemter AM, le Bihan T, Wear M, Hoving D, Filbey KJ, Hewitson JP, Henderson H, Gonzàlez-Cìscar A, Errington C, Vermeren S, Astier AL, Wallace WA, Schwarze J, Ivens AC, Maizels RM, McSorley HJ (2017). HpARI protein secreted by a helminth parasite suppresses Interleukin-33. Immunity.

[bib48] Pace F, Carvalho BM, Zanotto TM, Santos A, Guadagnini D, Silva KLC, Mendes MCS, Rocha GZ, Alegretti SM, Santos GA, Catharino RR, Paroni R, Folli F, Saad MJA (2018). Helminth infection in mice improves insulin sensitivity via modulation of gut Microbiota and fatty acid metabolism. Pharmacological Research.

[bib49] Panda AK, Ravindran B, Das BK (2013). Rheumatoid arthritis patients are free of filarial infection in an area where filariasis is endemic: comment on the article by Pineda et al. Arthritis & Rheumatism.

[bib50] Pandey P, Dixit A, Chandra S, Tanwar A (2015). Cytological diagnosis of bancroftian filariasis presented as a subcutaneous swelling in the cubital Fossa: an unusual presentation. Oxford Medical Case Reports.

[bib51] Pastille E, Frede A, McSorley HJ, Gräb J, Adamczyk A, Kollenda S, Hansen W, Epple M, Buer J, Maizels RM, Klopfleisch R, Westendorf AM (2017). Intestinal helminth infection drives carcinogenesis in colitis-associated Colon cancer. PLOS Pathogens.

[bib52] Pineda MA, McGrath MA, Smith PC, Al-Riyami L, Rzepecka J, Gracie JA, Harnett W, Harnett MM (2012). The parasitic helminth product ES-62 suppresses pathogenesis in collagen-induced arthritis by targeting the interleukin-17-producing cellular network at multiple sites. Arthritis & Rheumatism.

[bib53] Rajamanickam A, Munisankar S, Bhootra Y, Dolla C, Thiruvengadam K, Nutman TB, Babu S (2019). Metabolic consequences of concomitant Strongyloides stercoralis infection in patients with type 2 diabetes mellitus. Clinical Infectious Diseases.

[bib54] Rajamanickam A, Munisankar S, Dolla C, Menon PA, Thiruvengadam K, Nutman TB, Babu S (2020). Helminth infection modulates systemic pro-inflammatory cytokines and chemokines implicated in type 2 diabetes mellitus pathogenesis. PLOS Neglected Tropical Diseases.

[bib55] Rea IM, Gibson DS, McGilligan V, McNerlan SE, Alexander HD, Ross OA (2018). Age and Age-Related diseases: role of inflammation triggers and cytokines. Frontiers in Immunology.

[bib56] Rook GA, Adams V, Hunt J, Palmer R, Martinelli R, Brunet LR (2004). Mycobacteria and other environmental organisms as immunomodulators for immunoregulatory disorders. Springer Seminars in Immunopathology.

[bib57] Rzepecka J, Pineda MA, Al-Riyami L, Rodgers DT, Huggan JK, Lumb FE, Khalaf AI, Meakin PJ, Corbet M, Ashford ML, Suckling CJ, Harnett MM, Harnett W (2015). Prophylactic and therapeutic treatment with a synthetic analogue of a parasitic worm product prevents experimental arthritis and inhibits IL-1β production via NRF2-mediated counter-regulation of the inflammasome. Journal of Autoimmunity.

[bib58] Sanai SA, Saini V, Benedict RH, Zivadinov R, Teter BE, Ramanathan M, Weinstock-Guttman B (2016). Aging and multiple sclerosis. Multiple Sclerosis Journal.

[bib59] Shen SW, Lu Y, Li F, Shen ZH, Xu M, Yao WF, Feng YB, Yun JT, Wang YP, Ling W, Qi HJ, Tong DX (2015). The potential long-term effect of previous schistosome infection reduces the risk of metabolic syndrome among chinese men. Parasite Immunology.

[bib60] Smallwood TB, Giacomin PR, Loukas A, Mulvenna JP, Clark RJ, Miles JJ (2017). Helminth immunomodulation in autoimmune disease. Frontiers in Immunology.

[bib61] Sonnenberg GF, Fouser LA, Artis D (2011). Border patrol: regulation of immunity, inflammation and tissue homeostasis at barrier surfaces by IL-22. Nature Immunology.

[bib62] Strachan DP (1989). Hay fever, hygiene, and household size. BMJ.

[bib63] Tahapary DL, de Ruiter K, Martin I, Brienen EAT, van Lieshout L, Cobbaert CM, Soewondo P, Djuardi Y, Wiria AE, Houwing-Duistermaat JJ, Sartono E, Smit JWA, Yazdanbakhsh M, Supali T (2017). Effect of anthelmintic treatment on insulin resistance: a Cluster-Randomized, Placebo-Controlled trial in Indonesia. Clinical Infectious Diseases.

[bib64] Tchkonia T, Morbeck DE, Von Zglinicki T, Van Deursen J, Lustgarten J, Scrable H, Khosla S, Jensen MD, Kirkland JL (2010). Fat tissue, aging, and cellular senescence. Aging Cell.

[bib65] Teixeira AL, Wieck A, Diniz BS, Bauer ME (2012). Biomarkers in mood disorders among the elderly: can they contribute to diagnosis and prognosis?. Current Translational Geriatrics and Experimental Gerontology Reports.

[bib66] Thevaranjan N, Puchta A, Schulz C, Naidoo A, Szamosi JC, Verschoor CP, Loukov D, Schenck LP, Jury J, Foley KP, Schertzer JD, Larché MJ, Davidson DJ, Verdú EF, Surette MG, Bowdish DME (2017). Age-Associated microbial dysbiosis promotes intestinal permeability, systemic inflammation, and macrophage dysfunction. Cell Host & Microbe.

[bib67] Tsai C-Y, Shen C-Y, Liao H-T, Li K-J, Lee H-T, Lu C-S, Wu C-H, Kuo Y-M, Hsieh S-C, Yu C-L (2019). Molecular and cellular bases of immunosenescence, inflammation, and cardiovascular complications mimicking “Inflammaging” in Patients with Systemic Lupus Erythematosus. International Journal of Molecular Sciences.

[bib68] Turton JA (1976). Letter: ige, parasites, and allergy. The Lancet.

[bib69] Velasquez-Manoff M (2012). An Epidemic of Absence: A New Way of Understanding Allergies and Autoimmune Diseases.

[bib70] Vennervald BJ, Polman K (2009). Helminths and malignancy. Parasite Immunology.

[bib71] Wammes LJ, Hamid F, Wiria AE, May L, Kaisar MM, Prasetyani-Gieseler MA, Djuardi Y, Wibowo H, Kruize YC, Verweij JJ, de Jong SE, Tsonaka R, Houwing-Duistermaat JJ, Sartono E, Luty AJ, Supali T, Yazdanbakhsh M (2016). Community deworming alleviates geohelminth-induced immune hyporesponsiveness. PNAS.

[bib72] Wolfs IM, Stöger JL, Goossens P, Pöttgens C, Gijbels MJ, Wijnands E, van der Vorst EP, van Gorp P, Beckers L, Engel D, Biessen EA, Kraal G, van Die I, Donners MM, de Winther MP (2014). Reprogramming macrophages to an anti-inflammatory phenotype by helminth antigens reduces murine atherosclerosis. The FASEB Journal.

[bib73] Wu D, Molofsky AB, Liang HE, Ricardo-Gonzalez RR, Jouihan HA, Bando JK, Chawla A, Locksley RM (2011). Eosinophils sustain adipose alternatively activated macrophages associated with glucose homeostasis. Science.

[bib74] Xi H, Katschke KJ, Li Y, Truong T, Lee WP, Diehl L, Rangell L, Tao J, Arceo R, Eastham-Anderson J, Hackney JA, Iglesias A, Cote-Sierra J, Elstrott J, Weimer RM, van Lookeren Campagne M (2016). IL-33 amplifies an innate immune response in the degenerating retina. Journal of Experimental Medicine.

[bib75] Xia S, Zhang X, Zheng S, Khanabdali R, Kalionis B, Wu J, Wan W, Tai X (2016). An update on Inflamm-Aging: mechanisms, prevention, and treatment. Journal of Immunology Research.

[bib76] Zhang H, Xing Y, Kong H, Dai Y, He W, Ge S, Zhu YC (2012). Anti-atherogenic effect and its mechanisms of soluble egg antigen of *schistosomia japonicum* in ApoE-/- mice. Zhongguo Xue Xi Chong Bing Fang Zhi Za Zhi = Chinese Journal of Schistosomiasis Control.

[bib77] Zhuang Y, Lyga J (2014). Inflammaging in skin and other tissues - the roles of complement system and macrophage. Inflammation & Allergy Drug Targets.

[bib78] Zuo L, Prather ER, Stetskiv M, Garrison DE, Meade JR, Peace TI, Zhou T (2019). Inflammaging and oxidative stress in human diseases: from molecular mechanisms to novel treatments. International Journal of Molecular Sciences.

